# Incorporating genotype information in a precise prediction model for platinum sensitivity in epithelial ovarian cancer

**DOI:** 10.3389/fonc.2024.1461772

**Published:** 2025-01-07

**Authors:** Nai-Yi Du, Yan Li, Hui Zheng, Ya-Kun Liu, Lu-Sha Liu, Jianbang Xie, Shan Kang

**Affiliations:** ^1^ Department of Gynecology, the Fourth Hospital of Hebei Medical University, Shijiazhuang, Hebei, China; ^2^ Department of Molecular Biology, the Fourth Hospital of Hebei Medical University, Shijiazhuang, Hebei, China; ^3^ Department of Translation Medicine, Shijiazhuang Ninghong Biotechnology Co., Ltd., Shijiazhuang, Hebei, China

**Keywords:** epithelial ovarian cancer, drug sensitivity, predicting model, single-nucleotide polymorphism, clinical factors

## Abstract

**Objective:**

Develop a predicting model that can help stratify patients with epithelial ovarian cancer (EOC) before platinum-based chemotherapy.

**Methods:**

148 patients with pathologically confirmed EOC and with a minimum 5-year follow-up were retrospectively enrolled. Patients were classified into platinum-sensitive and platinum-resistant groups according to treatment responses. The correlation between clinical factors and drug sensitivity was evaluated using statistical tests. Approximately 700,000 single-nucleotide polymorphism (SNP) sites were assessed for association with drug sensitivity via the Genome-wide Association Study (GWAS). LASSO regression and manual selection were employed to reduce the number of variables. A predicting model based on optimized variables was constructed. The predictive ability of the model was assessed using the Kaplan-Meier curve.

**Results:**

No statistically significant association was found between clinical factors and drug sensitivity. Sixteen SNPs were preserved after the optimization. A predicting model for drug sensitivity was constructed based on those sixteen SNPs. Coefficients of the synergistic effect for each SNP were determined, and an algorithm of the Drug Sensitivity Index (DSI) was built. The DSI score can successfully distinguish the drug-sensitive or drug-resistant patients with sensitivity, specificity, positive predictive value, and accuracy of 94.7%, 83.3%, 90.8%, and 90.5%, respectively. In both the training set and validating samples, the Kaplan-Meier curve showed that the median PFS and mean OS were significantly differentiated between the predicted sensitive and resistant patients (*p*-value<0.001).

**Conclusions:**

A mathematical model incorporating genotype information could help predict the drug sensitivity of platinum-based chemotherapy before the treatment in EOC patients. A personal chemotherapy could be achieved based on the model.

## 1 Introduction

Ovarian cancer (OC) is the leading cause of death from gynecologic cancers in the world, and ninety percent of OCs are epithelial ovarian cancer (EOC) (1). Due to the lack of early symptoms and insufficient sensitive screening methods, most EOC patients are diagnosed at clinically advanced stages. Following the primary cytoreductive surgery, platinum-based chemotherapy is the first-line medication for advanced-stage EOC patients (2, 3). Although the majority of patients respond well to the treatment, approximately 15% of patients will show drug resistance at the initial stage of chemotherapy, and an additional 30% will relapse within six months after the treatment finished (4). Currently, almost all patients will receive platinum-based chemotherapy for the front line of treatment. Unfortunately, those platinum-resistant patients are deprived of their opportunity for early optimal intervention, such as non-platinum-based chemotherapy, targeted therapies, or immunotherapy. Therefore, patient stratification prior to the chemotherapy has a significant clinical impact.

Many clinical and genetic factors were reported to be influential on the sensitivity of EOC patients to platinum-based drugs. A study conducted by Winarno GNA et al. found that the mean age of the platinum-sensitive group was significantly lower than that of the platinum-resistant group (5). In addition, they observed that the stage of EOC may also affect the response to the chemotherapy. Ikeda et al. found that the residual tumor was an independent factor for the response to platinum (6). Regarding genetic factors, single nucleotide polymorphisms (SNPs) may be the main genetic factors responsible for the variations of drug response among individuals. Many researchers, including our group, have identified specific SNPs associated with drug sensitivity in EOC (7–12). Furthermore, other studies suggested that the serum level of lactate dehydrogenase and ratios of platelet/lymphocyte and neutrophil/lymphocyte can be used to predict the drug sensitivity of EOC patients (5, 6). Many models utilizing CT images alone, or using CT images with genetic factors, or combining CT images, clinical factors, and SNPs were also established to predict the sensitivity to platinum-based chemotherapy (13–15).

However, no consensus has been achieved on the best approach for predicting drug sensitivity. More importantly, a commonly encountered clinical problem was that two similar patients received identical chemotherapies but responded significantly differently. These divergent responses were most likely to be determined by genetics. In most previous reports, only one or a few SNPs were studied in one specific clinical setting. Limited SNPs usually were insufficient for an accurate prediction of the drug response, which is typically a multi-gene process (7–12, 15, 16).

In this study, we systematically assessed the effects of a large amount of SNPs and several clinical factors regarding the drug sensitivity of EOC patients. Based on the results, we built a mathematical model that could predict the patient’s response to platinum-based chemotherapy. The algorithm utilizes the genotyping information from 16 SNPS and can help gynecological oncologists stratify patients before the treatment.

## 2 Materials and methods

### 2.1 Study subjects

In this retrospective study, 148 EOC patients were recruited from the Fourth Hospital of Hebei Medical University between February 2008 and August 2016. The inclusion criteria for cases were as follows: i) Pathologically confirmed primary EOC at any age. ii) Clinically classified at stage III or IV according to the International Federation of Gynecology and Obstetrics (FIGO) criteria. No restriction on the histological subtypes of tumors. iii) After the standard primary cytoreductive surgery, the first-line treatment was the combination of paclitaxel and platinum; and the patient received the treatment for six to eight cycles. iv) The minimum follow-up period was five years or until the patient was deceased. The exclusion criteria included: i) Diagnosed as other types of cancers. ii) With a history of chemotherapy, radiotherapy, or immunotherapy before the surgery. iii) The patient received a non-standard primary cytoreductive surgery or a non-standard intravenous chemotherapy. The study was approved by the Ethics Committee of the Fourth Hospital of Hebei Medical University (2023KS119), and consent was obtained from all patients enrolled.

### 2.2 Surveillance and categorization of patients

The standard post-chemotherapeutical surveillances included serial physical examinations, the cancer antigen 125 (CA-125) blood test, and the computed tomography (CT) scanning as clinically indicated. According to the National Comprehensive Cancer Network (NCCN) guidelines, the recurrent disease was identified by comprehensive evaluation of supportive evidence such as clinical symptoms (i.e., pelvic pain and weight loss), blood biomarkers (i.e., elevated CA-125 levels), and imaging examinations (17). Patients who did not experience the relapse for not less than six months from the end of the last round of platinum-based therapy were defined as drug-sensitive, while patients who exhibited disease progression during chemotherapy or who experienced relapse within six months from the end of primary chemotherapy were considered to be drug-resistant (18). Progression-free survival (PFS) was defined as the time from the date of surgery to the first recurrence or the last follow-up. Overall survival (OS) was defined as the time from the diagnosis to the all-cause death.

### 2.3 DNA extraction and genotyping

The whole-blood sample (5ml) from each subject was collected and was used for the genomic DNA extraction as previously described by Miller et. al (19). DNA samples were quantified via spectrometry and were stored at −20°C in TE buffer. Genotypes of ~700,000 single nucleotide polymorphism (SNP) sites were determined by the Asian Screening Array system (Illumina, San Diego, CA, USA) following the manufacturer’s instruction.

### 2.4 Construction of predictive model

#### 2.4.1 SNP filtrations

All variants with a frequency of less than 0.05 or over 0.95 in the East Asian population were excluded, referring to the GnomAD v2.1.1 database (https://gnomad.broadinstitute.org/, last accessed on June. 26, 2024). The Genome-wide Association Study (GWAS) was performed to establish the association between the variant and the sensitivity to the platinum-based regimens. A cutoff value of *p <*0.05 was used to further downsize the number of initial inputting SNPs.

#### 2.4.2 Model developing strategy

The goal of our study is to build a mathematical model that can predict the sensitivity of epithelial ovarian cancer patients to platinum-based chemotherapeutic regimens. Although we believe that the sensitivity of EOC patients to the drugs is mainly determined by genetics, we cannot exclude the influence of non-genetic factors (age, stage, histology, and residual tumor). Firstly, we evaluated the correlation between the non-genetic factors and drug sensitivity using statistical tests. If any of these factors displayed a significant correlation with drug sensitivity, we would integrate that factor into our model. We also examined the genetic variants. Due to the significant number of genetic variants (genotypes), we first used the Least Absolute Shrinkage and Selection Operator (LASSO) regression to reduce the total number of potential variants, and we manually checked the biological functions of the gene loci containing those possible variants. Only variants that had meaningful functions to platinum or paclitaxel were preserved. Then, another round of linear regression was applied to establish the model. Of all 148 EOC patients, 104 data sets were randomly chosen for training purposes, and the remaining 44 sets were for validation.

#### 2.4.3 Initial assignment of SNPs

The genotype of each SNP site was transformed into a numerical value as its initial assignment. The human referential genome (version 37, GRCh37) was used as the standard and was referred to as the ‘wild type’. The most common alternative sequence from the dbSNP database was referred to as the ‘mutant’. A comparison was made between the patient’s genotype and the referential sequences. If the sequences of the two alleles were both the same as the wild type, the initial assignment for that SNP would be ‘0’. If at least one of the sequences of two alleles was the same as the mutant, the initial assignment for that SNP would be ‘1’. If at least one allelic sequence differed from either the wild type or the mutant, a value of ‘0’ would also be assigned to that site. The initial assigned value for SNP is an integer alternated as either 0 or 1.

#### 2.4.4 Model development

The predictive model was constructed based on the LASSO regression algorithm implemented in the R (version 3.5.1) package ‘glmnet’. The optimal regularization parameters were determined using a 10-fold cross-validation technique. Bootstrap analysis was utilized to sample the dataset with replacement 500 times. Subsequently, a model was constructed for each bootstrap cohort. The final model incorporated only markers that were observed in over 50% of all bootstraps.

### 2.5 Statistical analysis

Statistical analysis was performed with SPSS v24.0 for Windows (Chicago, IL, USA). The association between the patient’s characteristics and the drug responses was assessed by the Mann-Whitney U test for continuous variables (i.e. age) and the Fisher’s Exact test for categorical variables (i.e. Clinical histology). Survival curves were plotted by Kaplan-Meier analysis with the log-rank test. P < 0.05 was considered a significant difference.

## 3 Results

### 3.1 Characteristics of patients

As shown in **Table 1**, out of the 148 patients, 94 (63.5%) exhibited drug-sensitive, and 54 (36.5%) displayed drug-resistant. The median of PFS for drug-sensitive patients was 40 months, and the corresponding time for the drug-resistant patients was only seven months. The median of OS for drug-sensitive patients was 60 months, which was also significantly longer than that of the drug-resistant patients (22 months). In terms of histology, 92 (62.2%) out of the 148 patients were diagnosed with high-grade serous ovarian cancer, 18(12.1%) with low-grade serous ovarian cancer, and 38 (25.7%) with endometrioid ovarian cancer. There were 136 cases (91.9%) in FIGO stage III and 12 cases (8.1%) in FIGO stage IV. A total of 98 patients (66.2%) underwent satisfactory cytoreductive surgery, with 36 (24.3%) and 62 (41.9%) patients having residual tumor sizes of 0cm and 0-1cm, respectively. The remaining 50 patients (33.8%) underwent unsatisfactory cytoreductive surgery (residual tumor>1cm).

**Table 1:** The clinical characteristics of 148 EOC patients.

	Patients (n/%)	Median	Range
Age (years)			
<50	52 (35.1%)	45y	(34-49)y
≥50	96 (64.9%)	58y	(50-73)y
Clinical histology			
High grade serous	92 (62.2%)		
Low grade serous	18 (12.1%)		
Endometrioid	38 (25.7%)		
Stage			
III	136 (91.9%)		
IV	12 (8.1%)		
Residual tumor			
≤1cm	98 (66.2%)		
0cm	36 (24.3%)		
0-1cm	62 (41.9%)		
>1cm	50 (33.8%)		
Drug reaction			
Sensitive	94 (63.5%)		
Resistance	54 (36.5%)		
PFS of patients (months)			
PFS of sensitive		40m	(11-60)m
PFS of resistance		7m	(2-13)m
OS of patients (months)			
OS of sensitive		60m	(17-60)m
OS of resistance		22m	(5-53)m

### 3.2 Influence of clinical factors on the drug sensitivity

As shown in **Table 2**, no clinical factor was significantly associated with drug sensitivity among all tested non-genetic features such as age, clinical histology, stage, and residual tumor. Notably, in our dataset, 11 pairs of patients shared the same clinical characteristics including surgical methods, age, histological type, stage, chemotherapeutical regimen, and residual tumor status yet substantially differed in the drug sensitivity (**Supplementary Table S2**).

**Table 2:** Statistical comparison of the clinical parameters between sensitive and resistant patients.

	Patients with sensitive (n/%)	Patients with resistance (n/%)	P
Age (range/median)	(34-73) y/55y	(45-73) y/53y	0.8418
Clinical histology			0.2599
High grade serous	54 (58.7%)	38 (41.3%)	
Low grade serous	12 (66.7%)	6 (33.3%)	
Endometrioid	28 (73.7%)	10 (26.3%)	
Stage			0.2116
III	84 (61.8%)	52 (38.2%)	
IV	10 (83.3%)	2 (16.7%)	
Residual tumor			0.2075
≤1cm	66 (67.3%)	32 (32.7%)	
>1cm	28 (56.0%)	22 (44.0%)	

### 3.3 Association of genetic variants with the drug sensitivity

As aforementioned we could not find a clinical factor that affected the drug sensitivity significantly in our sample set. Consequently, we employed a whole-genome SNP array with approximately 700,000 loci to test the association of those SNPs with drug sensitivity. After the GWAS analysis, a total of 446 SNP loci were identified that exhibited potential associations (*p*-value <0.05) with the drug sensitivity. However, 446 SNPs cannot directly be used to predict drug sensitivity, and we constructed a mathematical model to downsize the number of SNPs for prediction.

### 3.4 Construction of the model(drug sensitivity index)

**Figure 1:**
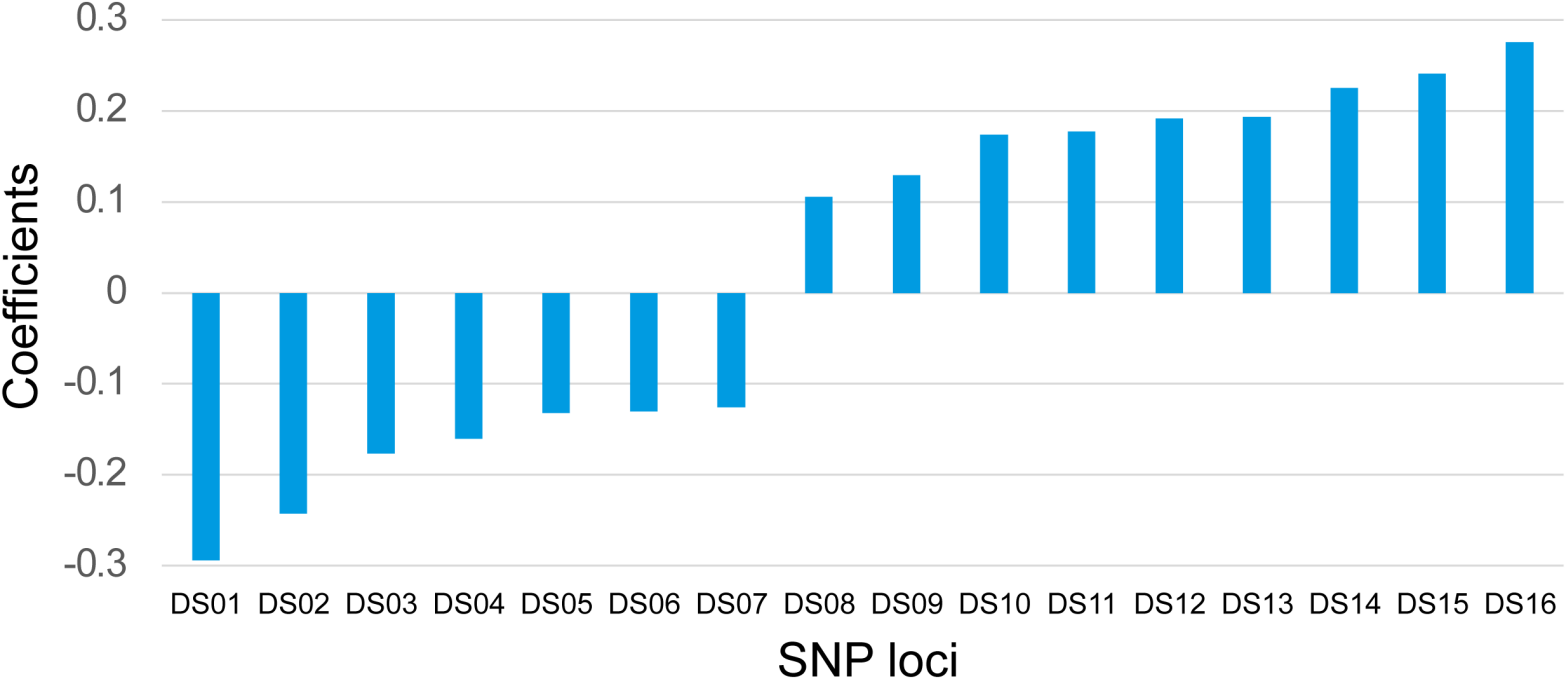
Coefficients of 16 SNP Loci. The coefficients of 16 SNP loci were shown for their synergistic powers on the prediction of the platinum-based drug sensitivity. X-axis: IDs of the SNP loci. Y-axis: Correlation coefficients.


Subsequently, we randomly divided 148 cases into the training set (104 cases, 70%) and the validation set (44 cases, 30%). The LASSO regression was applied to the 104 training datasets using 446 SNP loci. Briefly, each SNP site was initially assigned a value depending on its genotype (see method part). Next, the generalized linear model was applied using the R package. The nature of LASSO regression will automatically exclude non-significant variables. Then based on the remaining variables, we manually selected biologically meaningful SNP sites for the refinement. After the optimization, 16 effective SNP loci were selected for the predicting model. Among the 16 sites, seven had a negative coefficient, and nine were positive, with values varying from about -0·29 to about 0·27 (**Figure 1**). The exact locations of these 16 sites and their precise coefficients are shown in **Supplementary Table S1**. By combining the assigned value and coefficient values of each site, a Drug Sensitivity Index (DSI) was defined as: DSI=0.50194+ (Ai×Ci). Where: Ai=assigned value of the site i, which equals “0” or “1”, Ci = coefficient value of the site i, the detailed value for each site see **Supplementary Table S1**.

$$\begin{array}{c} DSI=0.50194+(-0.29405\times DS1value)+(-0.24243\\ \times DS2value)+(-0.17686\times DS3\;value)+\;\ldots \;+\;(0.27524\\ \times DS16\;value) \end{array}$$

**Figure 2:**
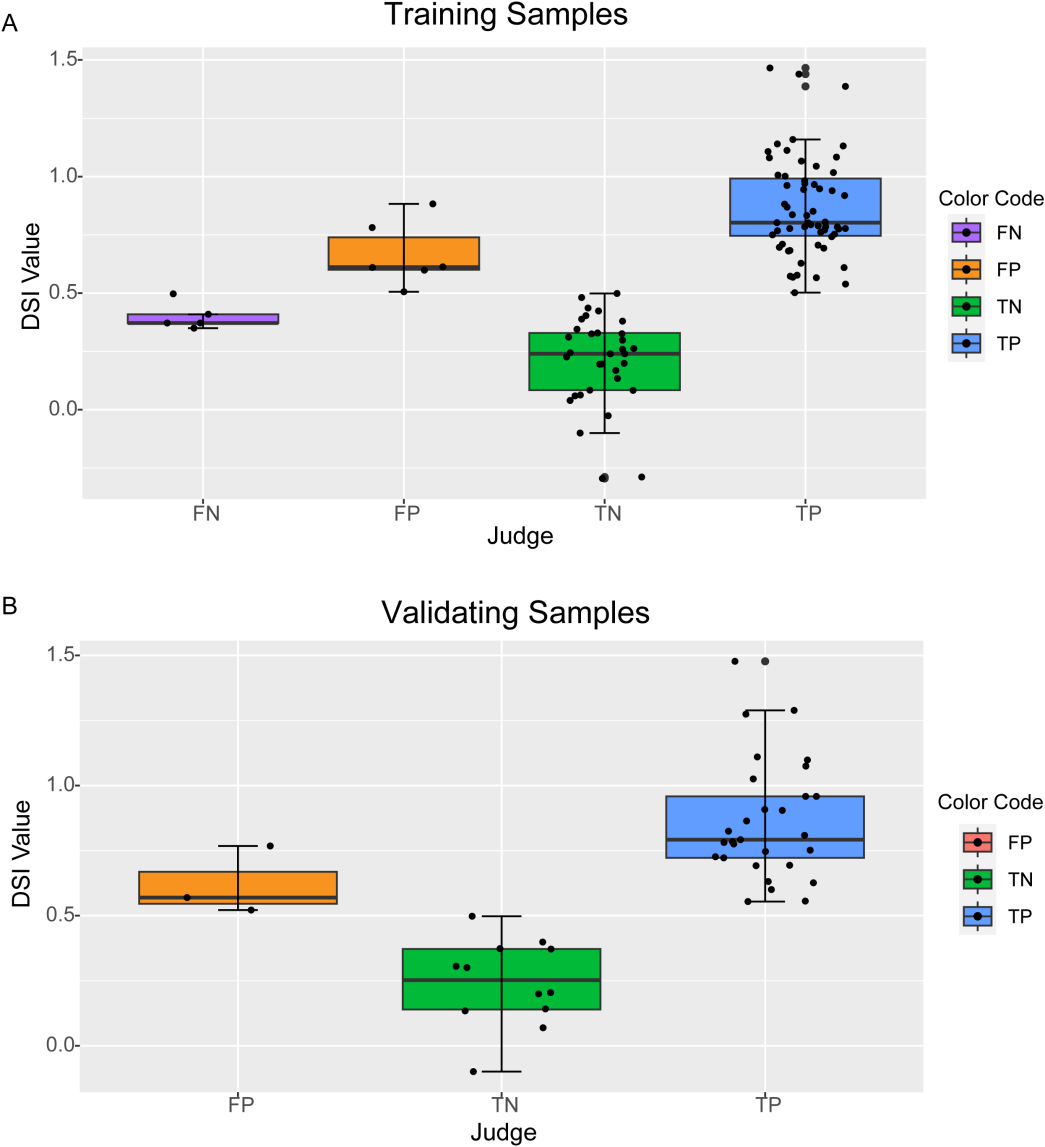
Performance of DSI in the training and validating samples. The DSI scores of each sample were calculated according to the LASSO regression model. The categories of prediction results were color indexed (FN= False Negative, purple; FP= False Positive, orange; TN=True Negative, green; TP=True Positive,blue). **(A)** Training samples. **(B)** Validating samples. X-axis: Prediction Results. Y-axis: DSI Scores.

The threshold of DSI was set to 0.5. If DSI ≥0.5 the patient would be predicted to be positive (drug-sensitive), and if DSI<0.5 the patient would be predicted to be negative (drug-resistant). We checked the training set by this threshold setting. There were 65 true drug-sensitive patients with five misclassified and 39 true drug-resistant patients with six misclassified (**Figure 2A**). The overall performance of this model on the 104 sets of training data was as follows: sensitivity, 92.3%; specificity, 84.6%; positive predictive value, 90.9%; and accuracy 89.4%.

### 3.5 Validation of the model

Based on this model, the DSI values were calculated for the 44 validating samples, and the results are illustrated in **Figure 2B**. 29 true drug-sensitive patients were all correctly classified and three of fifteen true drug-resistant patients were misclassified as drug-sensitive. The sensitivity, specificity, positive predictive value, and accuracy of the model for validating samples were 100%, 80.0%, 90.6%, and 93.2%, respectively. If the two sets of data were combined, the overall sensitivity, specificity, positive predictive value, and accuracy of the model were 94.7%, 83.3%, 90.8%, and 90.5%, respectively.

### 3.6 Evaluate the predictive ability of the model

**Figure 3:**
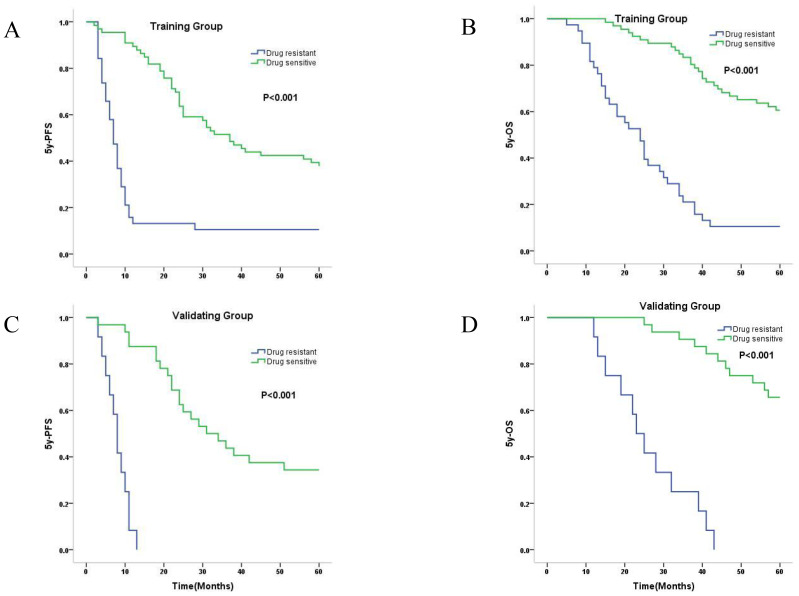
Kaplan-Meier survival curves of predicted drug-sensitive and resistant patients. **(A)** Median 5y-PFS of predicted drug-sensitive and resistant patients in training group were 37.0, and 7.0 months, respectively (P<0.001). **(B)** Mean 5y-OS of predicted drug-sensitive and resistant patients in training group were 50.7, and 25.8 months, respectively (P<0.001). **(C)** Median 5y-PFS of predicted drug-sensitive and resistant patients in validating group were 31.0, and 8.0 months, respectively (P<0.001). **(D)** Mean 5y-OS of predicted drug-sensitive and resistant patients in validating group were 54.0, and 26.0 months, respectively (P<0.001).

As shown in **Figure 3**, in both the training set and validating set, the Kaplan-Meier curve showed that the median PFS (**Figures 3A, C**) and mean OS (**Figures 3B, D**) were significantly longer for the predicted drug-sensitive patients compared to the predicted drug-resistant patients (PFS and OS in training group: 37m vs. 7m, 50.7m vs. 25.8m; PFS and OS in validation group: 31m vs. 8m, 54.0m vs. 26.0m). The log-rank test showed statistically significant differences in PFS and OS between these two predicted groups in both training and validating samples (all four P<0.001).

## 4 Discussion

In the present study, we investigated the clinical and genetic factors associated with platinum-based drug sensitivity in EOC patients. We established a regularized linear regression model incorporating 16 SNPs for predicting the drug susceptibility of EOC patients. The overall sensitivity, specificity, positive predictive value, and accuracy of the model were 94.7%, 83.3%, 90.8%, and 90.5%, respectively. The Kaplan-Meier curves showed the PFS and OS were significantly different in the predicted sensitive and resistant groups, indicating that the model has high predictive power for the EOC patients’ drug susceptibility.

Although Winarno G.N.A et al.’s study reported that the age and the FIGO stage were significant differences between the platinum-sensitive and platinum-resistant groups in EOC patients(P<0.05) (5), our study showed that there was no significant difference between the two groups regarding the age and staging (**Table 2**). It was consistent with the found of Asami Ikeda et al. that age was not a factor affecting the response to platinum-based drugs (6). We believe the divergence between our study and Winarno G.N.A, et al.’s work was caused by the FIGO staging difference of included patients. In their work, the studied patients were FIGO stage I to III EOC, and 42.2% of patients presented with stage I in the platinum-sensitive group. But in our study, there were no stage I or II patients. Similarly, Buttitta F. et al. included only stage III and IV patients in their study, and they did not find a significant staging difference between the platinum-sensitive and resistant group either (20), which was in line with our result. In our study, we also found there was no significant difference between the two groups in histology types (**Table 2**), which was supported by other reports (5, 6, 21). As for the residual tumor, the previous multivariate analysis showed that the residual tumor was an independent predictor of platinum resistance. Compared to patients with the absence of macroscopic residual tumor(R:0cm), those with macroscopic residual tumor (R:0-1cm and >1cm) exhibited an increased risk of developing platinum resistance (HR:2.111, 95%CI: 1.415-3.151, p<0.001) (6). Another study reported that compared to patients with residual tumor <2cm, those with residual tumor >2cm had an elevated risk of developing platinum resistance(OR:13.455, 95%CI: 2.624-68.992, p=0.0018) (20). However, our study showed that there was no significant difference between platinum-sensitive and resistant groups in residual tumor factor (**Table 2**). One possible explanation for this disagreement could be the differences in the criteria employed by different studies to group residual tumors. We adopted a grouped standard that closely aligns with clinical practice, which was whether a satisfactory cytoreductive surgery was conducted(R ≤ 1cm or R>1cm). This classification criterion was consistent with that utilized by Mendiola M., et al. (22). Another important factor is that although the extent of residual tumor should be documented clearly by the operating gynecological surgeon, it is recognized that there are limitations to the accurate recording of residual tumor, in both the measurement of lesions and the quantification of the tumor residuum (23). The personal subjective factors of an operating gynecological surgeon may affect the accurate recording of residual tumors.

From the above results, we found that the clinical factors (age, stage, residual tumor, and histology types) may not exert a dominant influence on the patient’s susceptibility to platinum-based chemotherapy. Furthermore, in our dataset, 11 pairs of patients shared identical surgical methods, age, histological type, stage, chemotherapy regimen, and residual tumor status, yet their drug responses differed significantly, which strongly implied that the inter-individual genetic variants may exert a significant influence on drug sensitivity. The most prevalent genetic variation in the human genome arises from single nucleotide polymorphisms (24–26). Previous studies have identified specific SNPs associated with the response to platinum-based chemotherapy in EOC (7–12). However, these studies focused only on very few variants and lacked a comprehensive assessment of the synergistic effect of multiple variants. The isolated results from these studies could barely be used for clinical guidance while the complexity of the human genome brought many difficulties for solving the problem methodologically. To overcome this drawback, the whole-genome-wise SNP array was employed in this study to comprehensively investigate approximately 700,000 SNP sites within the human genome. The goals of the study were to: 1.) discover as many as possible variants that are relevant to the drug sensitivity; 2.) quantitatively determine the effects of these effective variants; 3.) build an accurate model for the drug sensitivity prediction that can be easily used by the gynecological oncologists in the clinic. Although the epigenetics may also impact the chemotherapy resistance in EOC (27), we did not compare our discoveries with epigenetics. Because in terms of platinum resistance in ovarian cancer, there are two types of resistance: primary and acquired. The primary resistance means the patient showed resistance at the early stage of the platinum therapy (less than 6 months) and acquired resistance means the resistance shows up after a long period (over 12 months) (28). Generally, the primary resistance is determined by genetic variants and the acquired resistance is affected by alterations in multiple signaling pathways and usually is related to epigenetic modifications. Our study focuses on the primary resistance; therefore, we did not compare our discoveries with epigenetics.

One common problem for model construction is that the observed sample size (n) is much smaller than the number of predictive variables (p), and usually the variable selection (feature selection) is required. In our data, there is a small sample size (148 samples) and a high number of independent variables (about 700,000). It is obvious that n<<p. To solve this problem the LASSO regression was introduced by Robert Tibshirani in the year of 1996 (29). The LASSO regression is widely employed as a regularization method for linear models. As an alternative to the least squares regression, LASSO regression introduces an L1 penalty function as a regularization term, effectively addressing the problem of multicollinearity, promoting variable selection (feature selection), and helping to improve the predictive ability and interpretability of the model (30–33). In GWAS analysis, when multiple SNP loci are used as independent variables, the L1 norm-based feature selection can not only solve the problem of overfitting but also select important SNP loci (34). Combined with LASSO regression and biological feature selection, we successfully shrank the variables to 16 SNP sites. Then next round of least squares regression was applied. In this new scenario, the sample size (n) greatly exceeds the number of predictive variables (p), and the least squares regression worked well and showed highly accurate predicting capability (**Figure 3**). In the construction of the model, we also tested the elastic net regression which combined LASSO and Ridge regressions. However, the elastic net regression didn’t show any better performance when checked by the ROAUC curve and also resulted in more leftover variants. And we also tried other models including binary logistic regression. However, similar to the elastic net regression, the performance is no better than the LASSO regression.

In the manual selection, 16 SNPs were preserved based on their biological functions (**Supplementary Table S1**). 12 SNPs were located within the exonic region, while one each was found in the promoter, enhancer, 5’ UTR, and upstream regions. Notably, out of the 12 exonic SNPs, ten resulted in missense mutations, one led to a synonymous mutation, and another caused a nonsense mutation. When we searched the function of these 16 genes, three genes, including LIG1(DNA ligase 1), VEGFR2(one reception for VEGF), and PPP4R3A (a highly conserved member of the phosphatase family of serine/threonine phosphatases) have been demonstrated to be associated with the response to platinum and paclitaxel chemotherapy in EOC (35–41). Although the remaining 13 genes have not been conclusively associated with drug response in EOC they all have relevant bio-functions. The L3MBTL3, PLA2G7, KIAA1614, and C3orf33 genes were implicated in DNA repair, cell proliferation, apoptosis, and the cell cycle, and may be involved in the development of cancers (42–46). LAP3, ADRA1D, KIF25, GPS2, and MCF2L genes were associated with proliferation, movement, and invasion of tumor cells (47–52). CLEC7A and XIRP2 genes can contribute to cellular motility through their involvement with actin (53, 54). The CEP128 gene encodes a centrosomal protein that is a fundamental subdistal appendage protein and functions on the mother centriole for the organization of the centriolar microtubules (55, 56). Finally, the SLC25A39 gene was necessary for mitochondrial glutathione import in mammalian cells (57). Therefore, all those 16 SNPs may play some role in the pharmacology of platinum and paclitaxel combined chemotherapy.

Precision medicine, also known as personalized medicine, is a kind of medical care based on an individual’s genetic information and is the main direction of future medicine (58, 59). The fundamental concept for it is that each therapy should be customized based on personal characteristics. Genetic features are the most predominantly used personal characteristics for precision medicine. In clinical practice, both congenital genetic traits (e.g. germline mutations of breast and ovarian cancers) and acquired genetic traits (e.g. somatic mutations of lung cancer, etc.) have been employed in targeted therapy and immunotherapy interventions (60–63). In this study, we aim to develop a precision medicine approach that enables gynecologic oncologists to effectively utilize genetic features. Compared with other methods, our approach is relatively simplified and more clinically feasible. It just needs the blood sample, which can be easily obtained, and the genotype information, which can also be easily acquired either by fluorescent PCR, gene chip genotyping, or sequencing.

However, there were several limitations in our study. Firstly, the retrospective nature of this study may introduce selection bias. Secondly, the sample size in our study was modest, potentially impacting the stability and reliability of our model. Thirdly, this study is limited by its reliance on data from a single institution for both the training and validation cohorts. External validation using an independent cohort from other institutions will be necessary for the performance of our model. In future research, a prospective multicenter study should be conducted. Fourthly, we did not recruit any patients with other histotypes such as ovarian clear cell ovarian cancer in this study in addition to high-grade serous ovarian cancer, low-grade serous ovarian cancer and endometrioid ovarian cancer, although we did not restrict the histotypes in the inclusion criteria. Therefore, we restrict our conclusion within the histotypes included in our study. In future research, other histotypes may be concluded. Fifthly, the ethnic variances could compromise our conclusions in Western populations.

In summary, clinical factors may not exert a predominant influence on predicting the sensitivity of epithelial ovarian cancer to platinum-based chemotherapy, while a mathematical model that incorporates multiple genetic variations can achieve good outcomes in this context. Future prospective multicenter studies with a larger sample size should be conducted to validate our prediction model and to optimize it for clinical practice.

## Data Availability

The datasets presented in this study can be found in online repositories. The names of the repository/repositories and accession number(s) can be found in the article/**Supplementary Material**.
