# Corrigendum: Incorporating genotype information in a precise prediction model for platinum sensitivity in epithelial ovarian cancer

**DOI:** 10.3389/fonc.2025.1590905

**Published:** 2025-05-15

**Authors:** Nai-Yi Du, Yan Li, Hui Zheng, Ya-Kun Liu, Lu-Sha Liu, Jianbang Xie, Shan Kang

**Affiliations:** ^1^ Department of Gynecology, the Fourth Hospital of Hebei Medical University, Hebei, Shijiazhuang, China; ^2^ Department of Molecular Biology, the Fourth Hospital of Hebei Medical University, Hebei, Shijiazhuang, China; ^3^ Department of Translation Medicine, Shijiazhuang Ninghong Biotechnology Co., Ltd., Hebei, Shijiazhuang, China

**Keywords:** epithelial ovarian cancer, drug sensitivity, predicting model, single-nucleotide polymorphism, clinical factors

In the published article, there was an error in the **Funding** statement. “The author(s) declare financial support was received for the research, authorship, and/or publication of this article. This study was supported by Hebei Province Medical Science Research Key Project (No. 20230771)”. The correct **Funding** statement appears below.

“The author(s) declare financial support was received for the research, authorship, and/or publication of this article. This study was supported by Hebei Province Medical Science Research Key Project (No. 20230771) and the Natural Science Foundation of Hebei Province (No. H2020206535).”

The authors apologize for this error and state that this does not change the scientific conclusions of the article in any way. The original article has been updated.

