# Supplementary material for: Incorporating genotype information in a precise prediction model for platinum sensitivity in epithelial ovarian cancer
Source: Front Oncol. 2025 Jan 7;14:1461772. doi: 10.3389/fonc.2024.1461772 (PMC11746020; doi:10.3389/fonc.2024.1461772)
Supplement: Supplementary file 1 [file Table1.docx]

Supplemental Table 1: Coordinate of 16 preserved SNPs and corresponding genes

| **ID** | **Chr** | **Gene** | **Location** | **Coefficients** |
| --- | --- | --- | --- | --- |
| DS01 | 4 | LAP3 | 17586703 | -0.29405 |
| DS02 | 3 | C3orf33 | 155520356 | -0.24243 |
| DS03 | 6 | PLA2G7 | 46672943 | -0.17686 |
| DS04 | 14 | PPP4R3A | 91976351 | -0.16048 |
| DS05 | 14 | CEP128 | 81380682 | -0.13238 |
| DS06 | 12 | CLEC7A | 10282736 | -0.13024 |
| DS07 | 17 | GPS2 | 7216540 | -0.12547 |
| DS08 | 6 | KIF25 | 168442765 | 0.10558 |
| DS09 | 20 | ADRA1D | 4229218 | 0.12930 |
| DS10 | 6 | L3MBTL3 | 130374102 | 0.17381 |
| DS11 | 4 | VEGFR2 | 55979558 | 0.17710 |
| DS12 | 2 | XIRP2 | 168106246 | 0.19172 |
| DS13 | 19 | LIG1 | 48668830 | 0.19316 |
| DS14 | 1 | KIAA1614 | 180881148 | 0.22500 |
| DS15 | 17 | SLC25A39 | 42398052 | 0.24081 |
| DS16 | 13 | MCF2L | 113748872 | 0.27524 |

Referential Genome=GRCh37

|  |
| --- |
|  |
|  |
|  |
|  |
|  |
|  |
|  |
|  |

Supplemental Table 2 The clinical characteristics, PFS, and OS of 11 pairs of patients

| Group | Age(years) | Surgical method | Histological type | Stage | Chemotherapy regimen | Residual tumor | Drug reaction | PFS | OS |
| --- | --- | --- | --- | --- | --- | --- | --- | --- | --- |
| 1 | 45 | Primary cytoreductive surgery | High grade serous | Ⅲ | Platinum and palitaxel | ≤1cm | Sensitive | 11m | 34m |
| 1 | 45 | Primary cytoreductive surgery | High grade serous | Ⅲ | Platinum and palitaxel | ≤1cm | Resistance | 10m | 22m |
| 2 | 46 | Primary cytoreductive surgery | High grade serous | Ⅲ | Platinum and palitaxel | ＞1cm | Sensitive | 24m | 37m |
| 2 | 46 | Primary cytoreductive surgery | High grade serous | Ⅲ | Platinum and palitaxel | ＞1cm | Resistance | 5m | 15m |
| 3 | 48 | Primary cytoreductive surgery | High grade serous | Ⅲ | Platinum and palitaxel | ≤1cm | Sensitive | 19m | 47m |
| 3 | 48 | Primary cytoreductive surgery | High grade serous | Ⅲ | Platinum and palitaxel | ≤1cm | Resistance | 4m | 19m |
| 4 | 55 | Primary cytoreductive surgery | High grade serous | Ⅲ | Platinum and palitaxel | ≤1cm | Sensitive | 31m | 60m |
| 4 | 55 | Primary cytoreductive surgery | High grade serous | Ⅲ | Platinum and palitaxel | ≤1cm | Resistance | 8m | 43m |
| 5 | 56 | Primary cytoreductive surgery | High grade serous | Ⅲ | Platinum and palitaxel | ≤1cm | Sensitive | 51m | 60m |
| 5 | 56 | Primary cytoreductive surgery | High grade serous | Ⅲ | Platinum and palitaxel | ≤1cm | Resistance | 11m | 25m |
| 6 | 56 | Primary cytoreductive surgery | Endometrioid | Ⅲ | Platinum and palitaxel | ＞1cm | Sensitive | 60m | 60m |
| 6 | 56 | Primary cytoreductive surgery | Endometrioid | Ⅲ | Platinum and palitaxel | ＞1cm | Resistance | 10m | 38m |
| 7 | 56 | Primary cytoreductive surgery | Endometrioid | Ⅲ | Platinum and palitaxel | ≤1cm | Sensitive | 60m | 60m |
| 7 | 56 | Primary cytoreductive surgery | Endometrioid | Ⅲ | Platinum and palitaxel | ≤1cm | Resistance | 10m | 53m |
| 8 | 57 | Primary cytoreductive surgery | High grade serous | Ⅲ | Platinum and palitaxel | ≤1cm | Sensitive | 58m | 60m |
| 8 | 57 | Primary cytoreductive surgery | High grade serous | Ⅲ | Platinum and palitaxel | ≤1cm | Resistance | 9m | 26m |
| 9 | 59 | Primary cytoreductive surgery | High grade serous | Ⅲ | Platinum and palitaxel | ≤1cm | Sensitive | 21m | 60m |
| 9 | 59 | Primary cytoreductive surgery | High grade serous | Ⅲ | Platinum and palitaxel | ≤1cm | Resistance | 3m | 13m |
| 10 | 61 | Primary cytoreductive surgery | Low grade serous | Ⅲ | Platinum and palitaxel | ＞1cm | Sensitive | 14m | 37m |
| 10 | 61 | Primary cytoreductive surgery | Low grade serous | Ⅲ | Platinum and palitaxel | ＞1cm | Resistance | 8m | 20m |
| 11 | 65 | Primary cytoreductive surgery | High grade serous | Ⅲ | Platinum and palitaxel | ≤1cm | Sensitive | 60m | 60m |
| 11 | 65 | Primary cytoreductive surgery | High grade serous | Ⅲ | Platinum and palitaxel | ≤1cm | Resistance | 3m | 38m |
